# AMROBS: All-Metal Replicas of Biological Surfaces—A Novel Approach Combining Established Techniques

**DOI:** 10.3390/biomimetics3040031

**Published:** 2018-10-19

**Authors:** Florian Hischen, Mirjana Keser, Werner Baumgartner

**Affiliations:** Institute for Biomedical Mechatronics, Johannes Kepler University Linz, Altenberger Straße 69, A-4040 Linz, Austria; mirjana.keser@t-online.de (M.K.); werner.baumgartner@jku.at (W.B.)

**Keywords:** surface replication, galvanization, conductivity, surface modification

## Abstract

Biomimetic work often concerns to biological surfaces and their interaction with the environment. Liquid handling, barrier function and protection against heat, pathogens and predators, to name just a few, require biological surfaces to exhibit specific material properties—properties that often are not suited for specific measurements under lab conditions. In particular, the lack of the necessary sample toughness or conductivity can prove difficult to perform certain experiments. Hence, we present a novel approach to achieve all-metal replicas from biological surfaces (AMROBS). Resulting replicas exhibit microscale accurate replication of morphological topography while providing tough, conductive subjects for investigation and easy chemical surface modification. Combining established techniques like polymer casting (e.g., silicone), chemical silver precipitation and electroplating, all-metal replicas of several technical and biological surfaces (e.g., diffraction foils, lizard skin, flat bug surface) were produced and compared to their original counterparts with regard to morphology and functionality. By using scanning electron microscopy and video analysis, we show that a high degree of replication accuracy is achievable, and conclude the future possibilities of AMROBS in a comprehensive discussion, including the general “do’s” and “do nots” of metal replication following this approach.

## 1. Introduction

Biological surfaces—especially biomimetically interesting surfaces and (micro-) structures of animals and plants—have seen more and more scientific interest in the past few decades. Discoveries like the lotus effect [1,2], moisture harvesting in beetles [3], spider silk [4] and lizards [5,6], the interesting properties of butterflies’ wing scale structures [7,8] or the adaptive camouflage of bugs when in contact with rain [9] all have in common they consist on biological surfaces that exhibit an interesting phenomenon that scientists try to understand and transfer onto technical surfaces. Here, a common problem arises: morphological features of the biological antetype, which are responsible for the effect of interest, are often very small (within the nano- and micrometer range). Therefore, investigation becomes a challenge as it requires various forms of sample modification in order to prepare the biological material even for common methods like scanning electron microscopy (SEM) or transmission electron microscopy (TEM). Fixation and staining are necessary to enhance sample toughness and feature contrast. Additionally, biological samples often do not (or too faintly) exhibit a desired material based feature like thermal conductivity (e.g., human skin with a conductivity value of only 1–3 W m^−1^ K^−1^ [10], compared to copper with around 400 W m^−1^ K^−1^ [11]). This becomes problematic when investigating phenomena like the abovementioned adaptive camouflage on flat bugs and moisture harvesting, which are suspected to be influenced by conditions that lead to condensation. Since condensation is a phenomenon that involves relative ambient values of pressure, temperature and humidity with regard to a given surface, samples that exhibit the natural surface morphology but adjustable thermal conductivity are desirable for testing the theory.

Replication of just the morphological surface features is a task that generally can be achieved by various techniques. In simple cases, the surface is scanned (e.g., by SEM or atomic force microscopy (AFM), inferometric methods, laser or mechanical scanning), and in a digitally aided abstraction process, the relevant surface features are reduced to a required minimum. Afterwards, the features can be introduced into a desired material using a fitting manufacturing process. Casting, three-dimensional (3D) printing, lithography, laser etching or micromilling in technical surfaces like polymers and metals are just a few commonly used procedures. Successful examples for this kind of processing are various [12,13,14,15], however they all share a certain loss of detail in the final product—usually due to the reduction of information in the abstraction process or technical limitations. Such replicas can be good enough (and reasonable with concern to production cost and effort), however in many cases a true 1:1 copy is needed. To achieve this, in many cases the detailed imprinting capabilities of polymers are used to generate molds of a certain surface (e.g., [16,17,18]). Afterwards, these can be used for casting with a desired polymer (like resins or plastics) [19]. Such replicas show excellent replication detail and have been successfully used in the past to create precise copies of biological samples, and ready-to-use kits have reached the market (e.g., President SEM replication kit for high-resolution SEM replicas, Coltene/Whaledent, Switzerland). The chemical and mechanical material properties in these cases obviously depend on the chosen casting material. However, after testing different methods and materials, as well as screening the literature, we found no established replication technique that satisfied our demands with regard to material properties and simplicity. Investigating any influence of condensation phenomena in the liquid handling requires such samples to be (thermally) conductive in order to be able to quickly adjust sample temperatures in an environmental testing chamber. Hence, the all-metal approach was chosen.

Here, we report a novel approach to generate all-metal replicas of biological (and technical) surfaces, in short named AMROBS. These replicas, due to their metallic nature, have the benefits of sturdiness and durability, exhibit excellent conductivity values and still copy the target surface to maximum detail.

## 2. Materials and Methods

### 2.1. Sample Selection

Three different surfaces of both technical and biological origin were initially chosen as candidates for replication in this work: (1) diffraction grid foils; (2) the scaly skin of moisture harvesting desert lizards (in this case, the Texas horned lizard, *Phrynosoma cornutum*); and (3) the delicate surface of flat bugs (*Dysodius lunatus*), which live in rain forests and change their appearance depending on the wetting state. These three sample surfaces were chosen for their complexity and morphology (i.e., foils being the least complex and flat bugs being the most complex to replicate) and their relevance with regard to producing usable test subjects for eventual condensation testing (lizard surface and flat bugs) in line with our work. Lizard samples were ethanol-fixed museum specimen (Zoologisches Forschungsmuseum Alexander Koenig (ZFMK), Bonn, Germany). Flat bug samples were air-dried museum specimen (Tiroler Landesmuseum, Innsbruck, Austria).

### 2.2. The Replication Process

**Figure 1:**
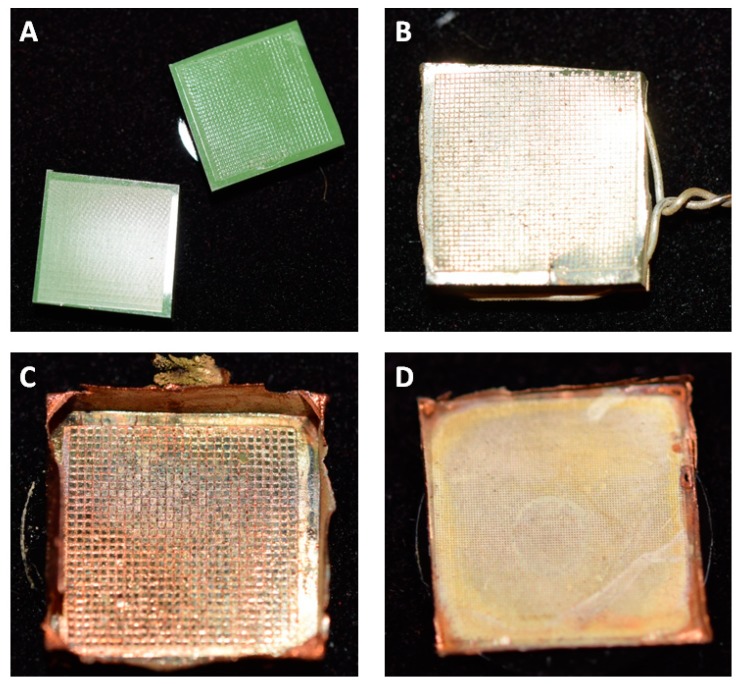
Replication process overview. (**A**) Silicone imprints of surfaces. (**B**) After silver precipitation, a thin layer of silver forms on top of an imprinted structure. (**C**) All-metal positive after electroplating with copper and removal of the silicone mold. The visible, structured surface is a silver–copper matrix copy of the originally imprinted surface. (**D**) All-metal replica after cleaning and gold coating. All samples are approximately 1 cm^2^ in size.

The general methodology for replicating any surface that was used in this work can be summarized as follows: (1) silicone imprinting to achieve a negative mold of the desired surface; (2) covering the silicone mold with a thin layer of precipitated silver (i.e., providing a conductive surface foundation); and (3) galvanizing (electroplating) the silver layer with copper in order to achieve a rigid, conductive structure. Afterwards, the replicas were separated from the molds and were post-processed if necessary (filing/sanding off any edges). Finally, they were covered by a nanolayer of gold (sputter coating) in order to stop oxidation processes of the surface. The individual steps of the process are visualized in Figure 1.

#### 2.2.1. Imprinting and Mold Casting

Negative molds of the desired surfaces were achieved by silicone imprinting using dental silicone (President JET Plus, light body, Coltene/Whaledent, Altstätten, Switzerland). Dental molding silicone was chosen because it is a non-toxic, quick curing ready-mix that has been successfully used to obtain highly detailed imprints of biological surfaces (e.g., [17,19]) and is already commercially available. Polymer and hardener were applied approximately 7 mm thick onto the target surface using a mixing syringe tip that comes with the silicone applicator. Then, the silicone puddle was lightly pressed with a flat surface (e.g., a plastic Petri dish) to achieve a final silicone thickness of 3–5 mm as well as a smooth bottom surface of the mold. This process worked well on rigid surfaces like the grid foils and the lizard skin. For delicate surfaces, such as the flat bugs, a sufficiently big puddle of silicone was placed on a flat plastic surface and the flat bug was carefully pressed into the silicone with a gloved finger. We found this approach to be sufficiently harmless to not deform or damage any of the used biological surfaces while molding, as we could not detect any faults in the finished replicas that would indicate that surface damage had been caused by the imprinting process. In one case, the wings of a dried bug specimen were cracked from the molding process and we found some waxy residue (detached from the animal surface) in the molds. Overall, the biological samples were intact after imprinting, especially the lizard skin and insects that were not too dry (old). After a curing time of approximately 5 min, the sample surface and silicone imprint were detached. The resulting silicone negatives (molds) were washed before further processing: the molds in contact with the biological material were washed for 5 min in boiling 10% KOH solution (≥85%, p.a., pellets, Carl Roth GmbH & Co. KG, Karlsruhe, Germany) in order to eliminate any biological residue (e.g., surface waxes and contaminations), followed by extensive rinsing in distilled water and 10 min washing in a ultrasonic bath (Bandelin Sonorex RK31, Bandelin electronic GmbH & Co. KG, Berlin, Germany); molds in contact with technical surfaces were washed in 80% ethanol for 10 min in the super-sonic cleaner. Surfaces that were imprinted include diffraction foils (grid sizes 1–2 µm distance between rows, products 01505-DG-500 and 01504-DG-1000, Rainbow Symphony Store, Reseda, CA, USA), the skin of the Texas horned lizard *P. cornutum*, and the surface of South American flat bugs *D. lunatus*. All animals that were used for these experiments were dead and were preserved specimens from collections. An exemplary step-by-step of the imprinting procedure is shown in Supplementary Figure S1.

#### 2.2.2. Silver Precipitation (Tollens Reaction)

A thin layer of silver was precipitated onto the cleaned silicone molds by means of Tollens reaction (as described elsewhere [20,21]), which was originally designed for the detection of aldehydes. In the solution, α-d-glucopyranose and β-d-glucopyranose are in equilibrium, and a small portion of the glucose exists in its open-chain aldehyde form (mutarotation). Mixing glucose and Tollens’ reagent results in the oxidation of glucose (in aldehyde form) into its corresponding carboxilate salt. The Tollens reagent itself consists of an ammoniacal silver nitrate solution, that is usually created by mixing a sufficient amount of ammonia to a slightly basic solution of silver nitrate. As a result, pure silver is precipitated. In this work, a fixed protocol of Tollens reaction was established in order to achieve reproducible silver films on samples. First, 1 g of silver nitrate (Sigma-Aldrich, Taufkirchen, Germany), was dissolved in 10 mL of distilled water, and ammonia (32% solution, Carl Roth GmbH & Co. KG) was carefully added until the solution cleared. Then, 0.15 g of ammonium sulfate (Sigma-Aldrich) was added to this solution and was filled up with distilled water to a total of 50 mL. Additionally, 25 mL of a KOH solution (18 g L^−1^) (≥85%, p.a., pellets, Carl Roth GmbH & Co. KG) and 25 mL of a 2.5% glucose (d-(+)-glucose, Carl Roth GmbH & Co. KG) solution were prepared. The solutions were mixed in the following order: (1) KOH solution; (2) glucose solution; and (3) ammoniacal silver nitrate solution. Dental silicon molds, wrapped with silver wire, were attached to the side of the beaker using tape in such a fashion that the mold was well covered with the reagent. The silver wires provided hanging support in this step, however they are necessary as a conductive connector in the following galvanization treatment. Precipitation took place under stirring on a magnetic stirrer for approximately 2.5 min. Success of the reaction was confirmed by a silver mirror precipitate that covered the glass of the reaction container. For each sample, the whole reaction was repeated four times in total in order to achieve an even, thin layer of precipitated silver on the samples. The samples were then carefully rinsed with distilled water to eliminate spontaneously formed carboxylated aldehyde salt crystals. Samples were dried for at least 24 h at ambient conditions before further treatment was performed (e.g., electroplating). The silver precipitation process is depicted in Supplementary Figure S2.

#### 2.2.3. Electroplating (Galvanizing)

Galvanization was performed in a custom setup as depicted in Supplementary Figure S3. Silver-covered silicone molds (cathode) were wrapped with a silver wire and were connected to the negative pole of a direct current (DC) power supply (B&K precision 9130, B&K Corp., Yorba Linda, CA, USA) and placed in a commercial copper sulfate electroplating solution (Conrad Elektronik, Hirschau, Germany). A piece of copper sheet metal was connected to the power supply’s positive pole (anode) and was arranged in the electroplating solution in such a way that the wide side of the piece was facing the silicon mold(s).

For the actual plating, voltage and amperage needed to be adjusted individually for each experiment. Using 3.0–3.2 V and 50 mA cm^−2^ produced consistent results during testing, however, for 3D samples, the total conductive area therefore needed to be calculated carefully and individually. In order to determine the copper deposition rate, six steel cubes of 1 cm^3^ each (custom manufactured from tool steel rods) were coated for 30 min. Afterwards, deposition was measured by weighing. Additionally, four copper sheet metal squares (1 cm^2^, 1 mm thick) were prepared and copper was deposited via electroplating onto each of them (2 h deposition time, copper-on-copper deposition). Cutting through these samples revealed the thickness of the deposited copper (see Supplementary Figure S4) and provided additional insight towards the deposition rate.

#### 2.2.4. Sputter Coating (Gold)

The finished copper replicas were sputter coated with a thin layer of gold (1) to improve contrast in the electron microscope; (2) to have chemical uniformity on top of the silver–copper matrix after electroplating and to prevent oxidation; and (3) to have an easy-to-modify surface ready for further chemical treatment (not shown in this work). Gold sputter coating was performed using a Polaron E5200 auto-93 coating device (Quorum Technologies, former Polaron Unlimited, Lewes, UK) at 1 kV for a duration of 180 s.

#### 2.2.5. Scanning Electron Microscopy and Video Analysis

Scanning electron microscopy images were obtained using a Philips SEM 525 (Philips, Hamburg, Germany) at 15 kV and working distances between 1 and 1.5 cm. Videos and photos were obtained with a Nikon D5300 digital camera (Nikon Corp., Minato, Japan) with an attached 60 mm macrolens (AF-S Micro 88 Nikkor 1:2.8, Nikon Corp.). Image post-processing was done by using GNU Image Manipulation Program (GIMP, version 2.10.6, https://www.gimp.org/).

## 3. Results

**Figure 2:**
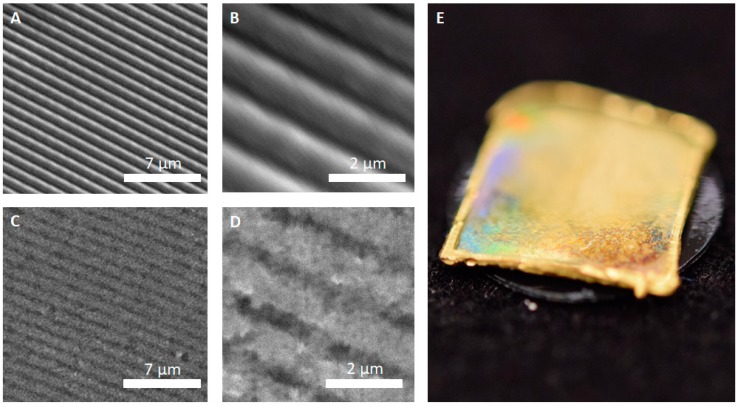
Comparison between the original grid foil and the copper replica. (**A**) Scanning electron microscopy (SEM) image of the grid foil showing periodic ripples with a width of 1 µm; and (**B**) higher maginification SEM image of the grid foil’s surface. (**C**) SEM image of the replica; and (**D**) higher magnification SEM image of the replica’s surface. (**E**) The surface of the all-metal replicated grid foil shows the iridescent light scattering that is typical for surfaces with periodic microstructures.

The first replicas were produced from diffraction grid foils. These foils exhibited highly regular, periodic microstructures—successful replication of their surface was the first step in evaluating the feasibility of the approach for delicate biological samples. A comparison between the original grid foil and the all-metal replica is shown in Figure 2.

Figure 2 shows that the individual grid lines of the foil can be successfully replicated into the copper–silver surface matrix of the replicas. Individual lines were cleanly copied (Figure 2C,D) and no apparent major defects were visible. In detail, however, the replica (Figure 2D) revealed a slightly grainier texture in comparison to the smoothness of the original. Still, as shown in Figure 2E, even though the surface is not as smooth as the original, the replica exhibited a rainbow like iridescence that is typical for periodically microstructured surfaces.

Next, in order to determine the copper deposition rate, we performed a series of verification trials. Standardized steel cubes and copper sheet metal squares were manufactured and galvanized according to the protocol above (galvanizing at 50 mA cm^−2^ and 3 V). The steel cubes (*n* = 6) were plated for 30 min, while the copper sheet metal samples (*n* = 4; 1 cm^2^, 1 mm thick) were plated for 2 h. Weighing the cubes after galvanization revealed the deposition rate, while the sheet metal squares were cut in half with a small diamond cutting disc (Dremel cutting disc Ø 22 mm, Dremel, Racine, WI, USA) to allow for measurement of layer thickness and the evaluation of connection quality in between the two coating layers.

As a result, we found that the steel cubes gained 0.175 ± 0.02 g after 30 min of plating, which translated into 63.8 ± 7.7 µm h^−1^ of layer growth. Measurements of the cross-cut thickness of “Layer 2”, shown in Supplementary Figure S2A,B, revealed a thickness of 129.4 ± 22.1 μm after 2 h deposition (*n* = 10 for each of the four manufactured samples). This translated into a deposition rate of 64.7 ± 11.05 µm h^−1^ and, therefore, confirmed the value found for the steel cubes.

**Figure 3:**
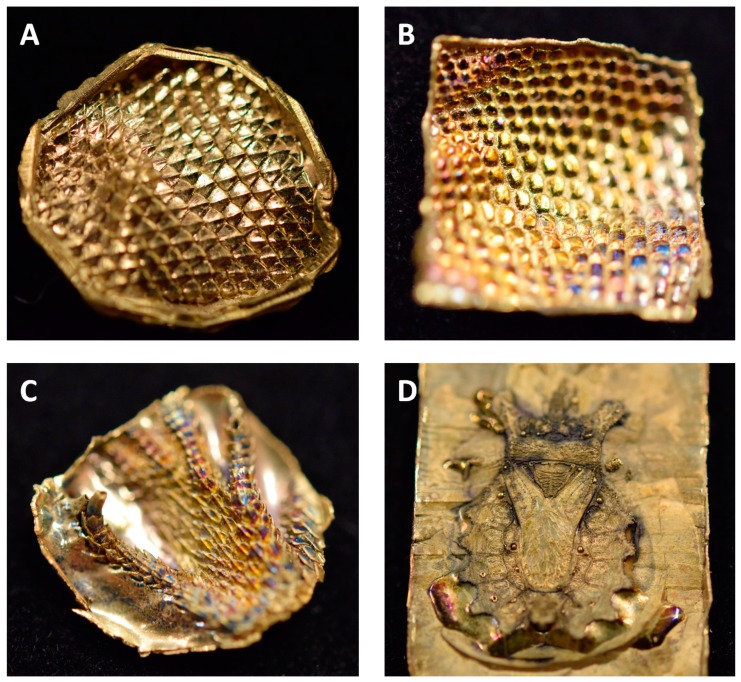
Representative examples of the all-metal replicas of biological surfaces. (**A**) Ventral and (**B**) lateral scales of *P. cornutum*. (**C**) Foot of *P. cornutum*. (**D**) Whole dorsal body surface of *D. lunatus*. Replicas shown in (**A**–**C**) are approximately 1 cm^2^, and replica shown in (**D**) is approximately 1 × 2 cm.

Replications of biological samples were produced specimen of Texas horned lizard (*P. cornutum*), as well as the dorsal surface of a flat bug (*D. lunatus*). For the lizard, silicone imprints of different body parts were selected in order to replicate the various kinds of scales that these animals exhibit. Figure 3 summarizes the exemplary achieved by the all-metal replicas technique.

**Figure 4:**
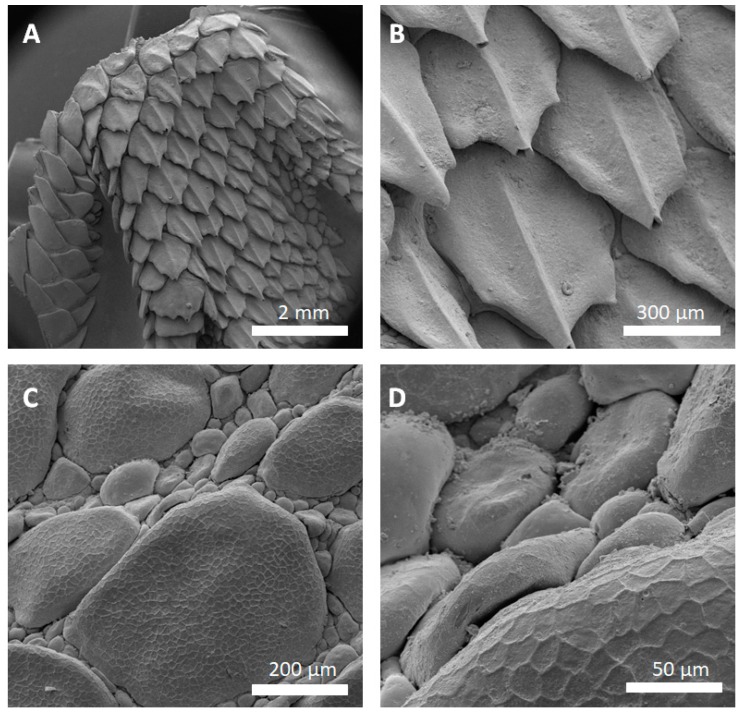
All-metal replica from the skin of *P. cornotum*. (**A**) SEM image of the foot scales’ replica; and (**B**) higher magnification SEM image. Minor defects as missing scale tips and small bubbles are observed. (**C**) SEM image of the dorsal scales’ replica; and (**D**) higher magnification SEM image. Successful replication of the lizards scales’ micro-ornamentation is clearly visible.

Figure 4 shows SEM images of successfully replicated skin regions of *P. cornutum*. All relevant skin features of the lizard were successfully replicated into the metal and the lizards’ surface microstructures were identified in the SEM images. Noteworthy, the honeycomb-like microstructure covering the lizards’ scales (as described in [5]), was clearly copied into the metal (Figure 4C,D). Figure 4A,B show that high aspect ratios and the undercuts are reproducible: the big, overlapping scales of the lizards’ foot were sharply replicated. Minor replication defects can be found in the gold-coated silver–copper replicas of the lizards’ foot (Figure 4A,B, tips of the scales are missing).

**Figure 5:**
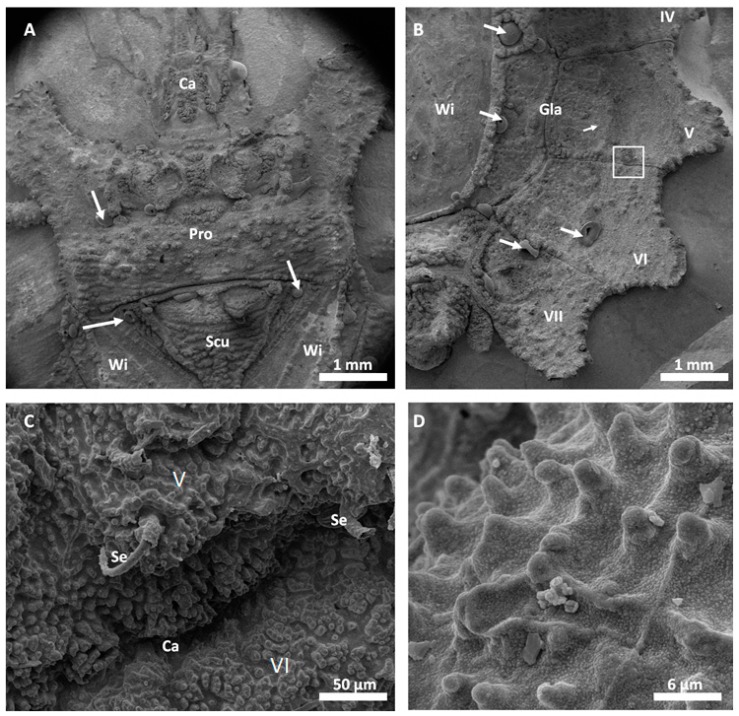
All-metal replica of *D. lunatus*. (**A**) SEM image of a replica of the dorsal–anterior part of *D. lunatus*. Morphological features can be identified: head (caput, Ca), pronotum (Pro), scutellum (Scu), and the sclerotized parts of the front wings (Wi). Bubble-like defects are indicated by white arrows. (**B**) SEM image of a replica of the dorsal–posterior part of the replica showing the connexival plates VI–VII with all of their corresponding intersegmental sutures, the glabrous area (Gla), and the wing membrane (Wi). Bubble-like defects can also be observed (white arrows). (**C**) Higher magnification SEM image from the white box in (**B**) shows he intersegmental suture between connexivum V and VI (capillary, Ca), the surrounding microstructured surface, and setae (Se). (**D**) Higher magnification SEM image of the surface microstructure.

**Figure 6:**
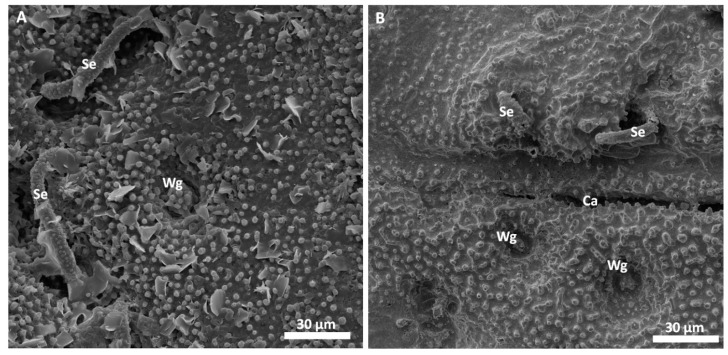
Comparison between (**A**) the original surface of a sputter coated specimen of *D. lunatus* and (**B**) an all-metal replica. The SEM images depict how well the all-metal surface replicates the natural counterpart. All relevant features are copied in detail: wax glands (Wg) and setae (Se), and surface microstructure. Crevasses of intersegmental capillaries (Ca) can also be replicated to a certain degree.

Scanning electron microscopy images of the successfully replicated dorsal surface of *D. lunatus* are shown in Figure 5 and Figure 6. These images revealed that all relevant morphological features of the flat bugs’ surface were replicated into the metal surface. Wings, capillaries, setae and the waxy microstructure of the integument were cleanly copied and resembled the morphology of the original surfaces as shown and described in [9]. The overall amount of surface defects in this replica was relatively low (bubbles, marked by white arrows).

**Figure 7:**
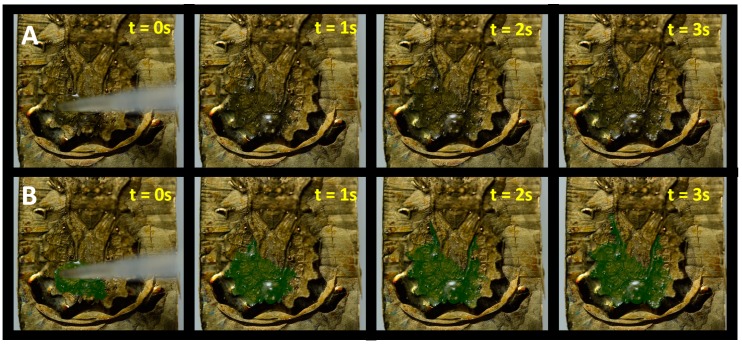
Oil spread on the dorsal surface of an all-metal replicated flat bug. (**A**,**B**) Frames from Supplementary Video S1 (seconds 12–16) show spread of oil on the replica surface. Oil is colorized in (**B**) for contrast and better visualization.

In order to test the functionality of the replicated falt bug surface, the wetting behavior was tested analog to the experiments we presented in [9]. On the surface of a real bug, water spreads fast in all directions, wetting the insect’s integument. Water transport hereby is facilitated by the surface chemistry, the microstructure and the capillary system in between the segmental plates. To account for the changed surface chemistry of the all-metal replica, we dropped small amounts of WD-40 multipurpose oil (WD-40 Company, San Diego, CA, USA) onto the replica and recorded how the oil spread. Supplementary Video S1 shows this experiment and Figure 7 summarizes the results. The use of WD-40 oil served no other purpose than to exemplarily show that the artificial surface of the replica is able to perform similar tasks as the natural antetype, however in a completely different chemical regime: on the strongly hydrophilic surface of the original flat bug, fluid spread with oil would never occur. Instead, a droplet of WD-40 oil would form a sphere and eventually roll off the surface. Additionally, WD-40 oil is a penetrating oil and exhibits low viscosity, which speeds up the spreading and improves the visual effect.

## 4. Discussion

The experiments and results reported here can be considered preliminary, and extensive testing with concern to reproducibility, functionality and actual degree of replication is planned for the future. However, the presented findings clearly suggest the overall feasibility of the AMROBS concept and the justification to continue to work in this direction. We achieved to copy delicate technical and biological surfaces into metal with a high degree of replication accuracy while allowing the replicas to offer all the benefits of the metallic material matrix: rigidity, durability and wear resistance, as well as excellent conductivity.

Replication of the technical surface (diffraction grid foil, as shown in Figure 2) yielded excellent results and the justification to attempt replication of biological samples. Following the AMROBS approach, all-metal replicas of different surface parts of lizard (*P. cornutum*) skin and flat bug (*D. lunatus*) could be fabricated (as shown in Figure 4, Figure 5 and Figure 6). The SEM images revealed the the described replication method to be successful: individual morphological features of the replicas’ natural counterparts were virtually copied 1:1. Features like the honeycomb-like microstructures on the scales of *P. cornutum* (Figure 4) were pronounced almost as sharply as on the natural skin, and even the fine and delicate wax microstructures of *Dysodius* exhibited the typical appearance (Figure 5C,D). In a direct comparison with the natural counterpart (Figure 6), one can clearly see how exact the surface was replicated into all-metal. Even shapes that exhibited fine and hollow features like setae (Figure 5C and Figure 6B) can be reproduced by this technique. This indicates that (1) the imprinting process using dental silicone is an appropriate way to mold very fine surface structures without destroying them; and (2) the subsequent surface coatings with silver and copper produce a matrix fine enough to infill these most delicate imprints in the mold.

However, the yielded metal surfaces were not completely void of smaller defects. As depicted in Figure 4B, as well as in Figure 5B, small, bubble-like artefacts occurred in the replicas. These were most likely the result of small enclosures of air during imprinting with silicone (and, therefore, result in the empty pockets that were getting filled with copper in the electroplating process). More dedicated testing towards this problem has to be addressed in the future, and a possible solution might be to execute imprinting and curing in an evacuated environment. Also, small empty spots occurred in the replicas, as exemplarily shown for the tips of the lizard scales (Figure 4B). Here, most likely deposition of the conductive material (silver) did not reach the deepest spots in the silicone mold. Consequently, no copper could be deposited there as the electroplating process filled the mold in a consecutive manner (hence, the tips of the scales were missing in the replica). To avoid future defects of this kind, it is suggested to choose the orientation of the molds in the silver precipitation bath carefully in order to avoid precipitation shadows. Also, achieving better control in the silver precipitation step will be key (i.e., controlling the temperature and, therefore, the reaction speed).

Despite the abovementioned defects, AMROBS samples will allow for carrying out a variety of desired experiments in the future. Thanks to the excellent thermal conductivity, experiments concerning wetting, evaporation and condensation will become possible: AMROBS samples allow for quick changing of sample temperature, for example, by the usage of proper sized Peltiere elements. In addition to that, the sturdy qualities of the all-metal replicas will allow for multiple usage of the same sample, a feature that not necessarily applies to the originals. Additionally, given the uniform surface chemistry, all-metal replicas can be used for individual experiments that require explicitly designed chemical surfaces. In case of *Dysodius*, for example, it is feasible to coat the metal replicas with thin layers of artificially designed waxes. This could be achieved by vacuum deposition and has already been successfully shown for technical replications of self-organizing waxes, biomimetically inspired by plant surfaces (e.g., [19]). Afterwards, the liquid–surface interaction of flat bugs, as presented in [9], would become reproducible and adjustable. Additionally, gold used as a surface finish (in the presented work mainly to prevent further oxidation) is an excellent starter for further modifying the surface chemistry in general: the mercapto head group of thiols is known to form complexes with gold, whereas the rest of the molecule can be designed and modified to individual needs to achieve a desired surface chemistry (e.g., [22,23]). Combining this feature with gold-sputtered copper replicas may allow for diverse experiments that require stable surface morphology and adjustable surface chemistry, as well as excellent thermal and electrical conductivities.

### Do’s and Do Nots of AMROBS

One of our main reasons for publishing the results in such an early stage is to present this novel but easy approach of replication to the broader audience. In our opinion, research with the need for conductive copies of particularly, however not only, biological samples might benefit from this knowledge with the chance of adjusting and verifying the process to individual needs. Therefore, some general rules and observations of our initial work with the AMROBS approach should be addressed.

We found that one of the most crucial steps in the whole process was the initial coverage of silicone molds with a conductive layer in order to enable electrochemical deposition in the following electroplating step. As numerous pretests showed, this layer could be achieved by different means, however the result can drastically differ. We tested different approaches to render a conductive surface, such as sputter coating or evaporation-based applications. These transfer a small layer of metal or conductive non-metal material (like carbon). In both cases, the outcome could indeed be galvanized, and all-metal replicas were generated, however for different reasons, the final result was undesirable: sputter coating formed a very good bond between the silicone and the matrix of sputtered and electroplated material. The more (micro-) structured a surface, the stronger the bonding. In many cases, this rendered detachment of the replica from the mold impossible. For evaporation-based methods (like cathodic arc evaporation of carbon), the problem was that most evaporation-based coating techniques heat up the silicone mold and cause it to expand. In this case, we could achieve a successful replication process, however we found that the surface features were changed. After coating and cooling down, the silicone shrunk and most coatings started to wrinkle and form fault lines. From our experience, these fault lines will override major parts of the original (micro-) structures.

## 5. Conclusions

The AMROBS approach indeed offers a very promising way of obtaining high quality replications of most surfaces while being inexpensive, reliable and easy to follow at the same time. The first prototype replication shown in this work suggests that the technique delivers what was initially intended for: artificial replicas of biological surfaces that exhibit excellent durability, conductivity and an option to adjust the surface chemistry as desired. Moreover, extensive testing is needed to verify baseline information about reproducibility, actual replication degree and quality, as well as long-term material behavior. Elaboration of the replication technique itself should and will involve the improvement of the replicas’ surface quality. Furthermore, the usage of different metals will be tested in order to check the potential of, e.g., different bronzes or gold alloys, as they are popular in the field of medical healthcare, to broaden the possibilities of the approach.

## Supplementary Materials

The following are available online at http://www.mdpi.com/2313-7673/3/4/31/s1, Figure S1: Imprinting process with dental silicone, Figure S2: Silver deposition via chemical precipitation, Figure S3: Custom electroplating setup, Figure S4: Representative SEM images of copper deposited on copper by electroplating, Video S1: Oil spread on all-metal flat bug surface replica.
