# Supplementary material for: AMROBS: All-Metal Replicas of Biological Surfaces—A Novel Approach Combining Established Techniques
Source: Biomimetics (Basel). 2018 Oct 19;3(4):31. doi: 10.3390/biomimetics3040031 (PMC6352663; doi:10.3390/biomimetics3040031)
Supplement: Supplementary file 1 [file biomimetics-03-00031-s001.pdf]

Biomimetics **2018**, *3*, 31

Supplementary Materials: AMROBS: All-Metal Replicas of Biological Surfaces—A Novel Approach Combining Established Techniques

Florian Hischen *, Mirjana Keser and Werner Baumgartner

Institute for Biomedical Mechatronics, Johannes Kepler University Linz, Altenberger Straße 69, A-4040 Linz, Austria; mirjana.keser@t-online.de (M.K.); werner.baumgartner@jku.at (W.B.)

* Correspondence: florian.hischen@jku.at

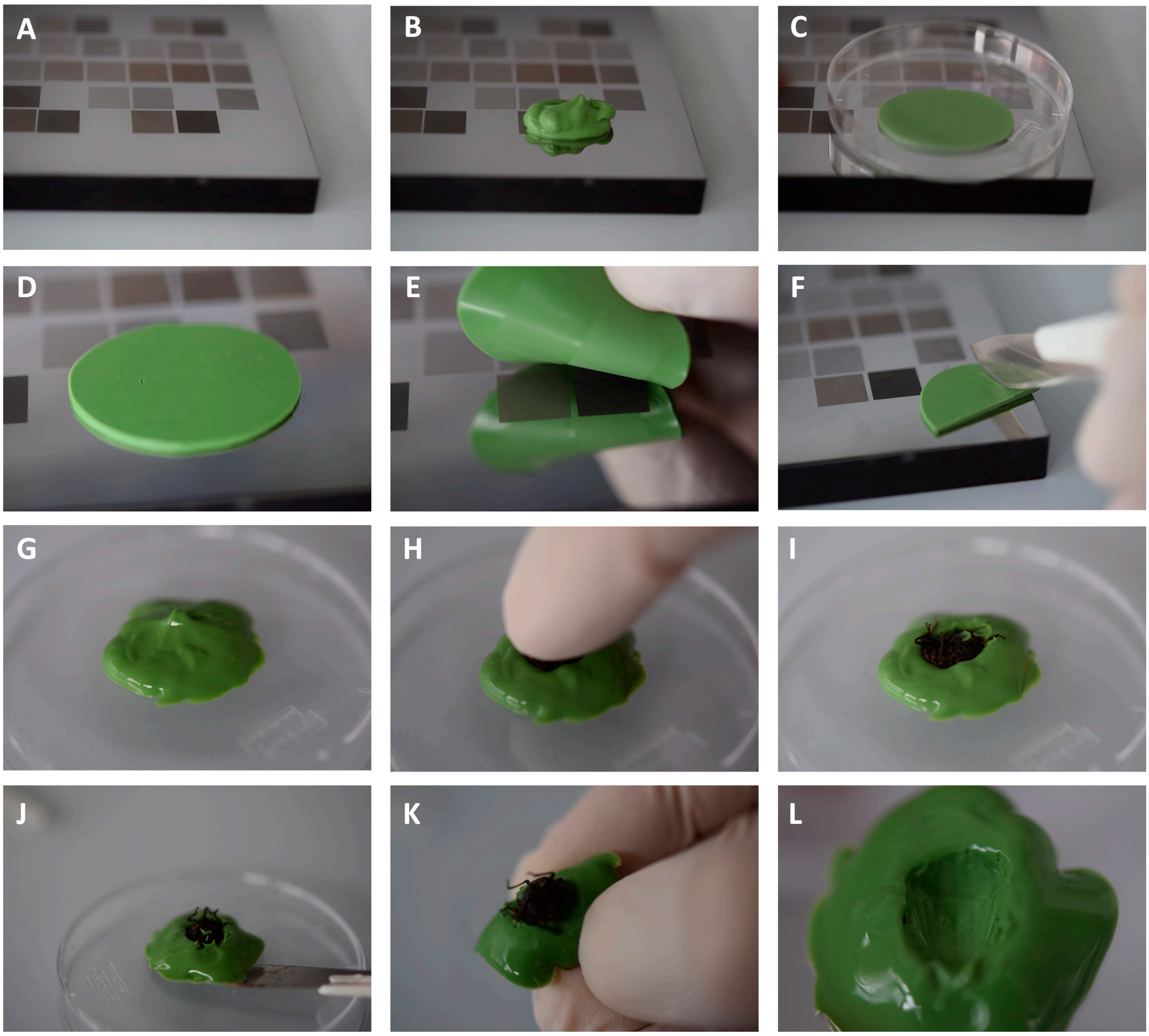

Figure S1. Imprinting process with dental silicone. (**A)** Steel plate containing laser engraved test fields of various microstructures (produced by Fraunhofer-Institut für Produktionstechnologie (IPT), Aachen, Germany, Aachen). (**B)** A drop of dental silicone is applied. (**C)** The smooth side of a plastic Petri dish lid is used to evenly flatten the silicone on top of microstructured fields of interest. (**D)** After approximately 5 min of curing time, the plastic cover can be detached and the sample can sit for a few seconds to ventilate. (**E)** The imprint can be extracted. (**F)** A scalpel can be used to shape and trim the samples. (**G)** For replications of biological surfaces, a drop of silicone is placed on a smooth plastic Petri dish lid. (**H**) The sample is immediately pressed into the silicone (in this case, a preserved specimen of *Graphosoma lineatum*). (**I**) Next, the silicone is cured for 5 min. (**J**) Lifting the silicone carefully from the Petri dish lid. (**K**) By bending the silicone, the sample will pop out easily (and usually undamaged). (**L**) The finished imprint is ready for silver deposition.

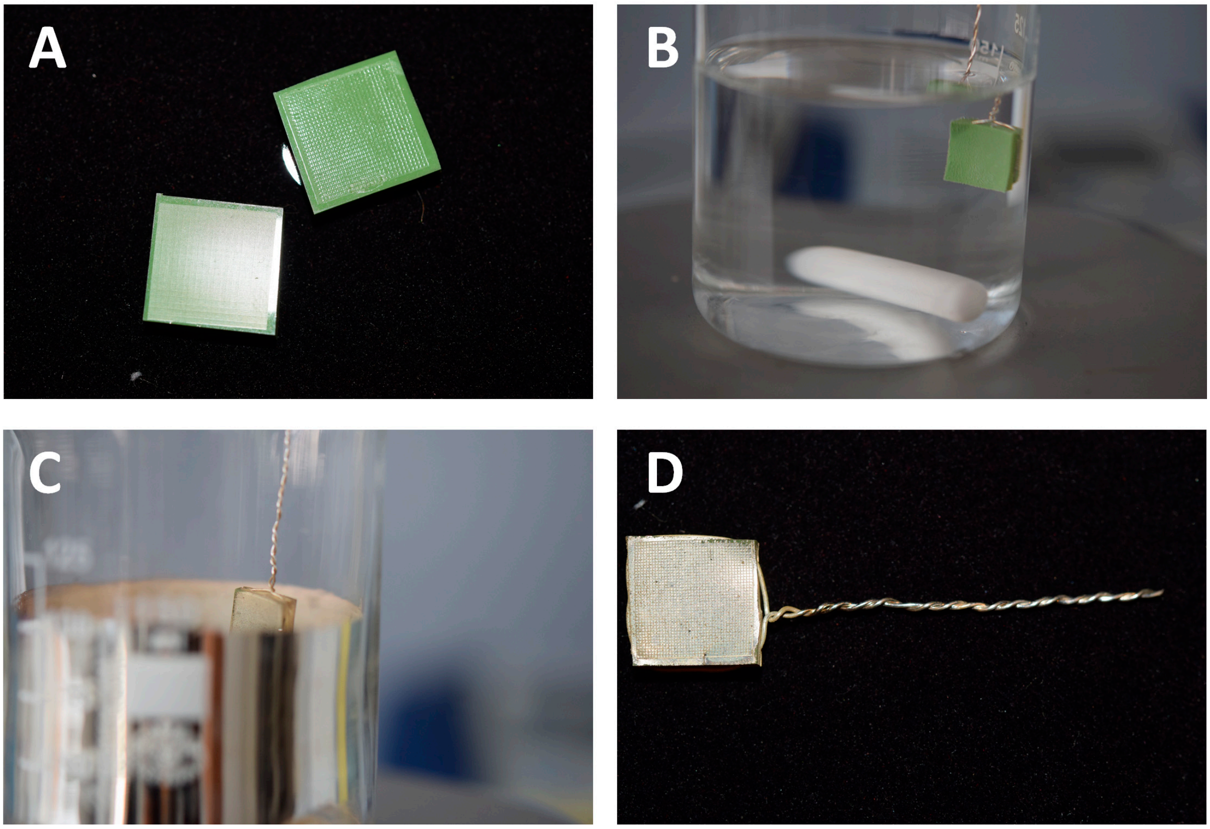

Figure S2. Silver deposition via chemical precipitation. (**A**) Silicone imprints are cut to the desired size and (**B**) are hung in a glass container using a silver wire. (**B**) Placing the sample close to the edge of the container and stirring while Tollens reaction occurs proved to be the most successful (in the depicted scene, the reaction has yet to be started by adding the last chemical). (**C**) Successful reactions lead to silver deposition on both the reaction container and the silicone imprint. (**D**) Finished sample after four repetitions of silver precipitation.

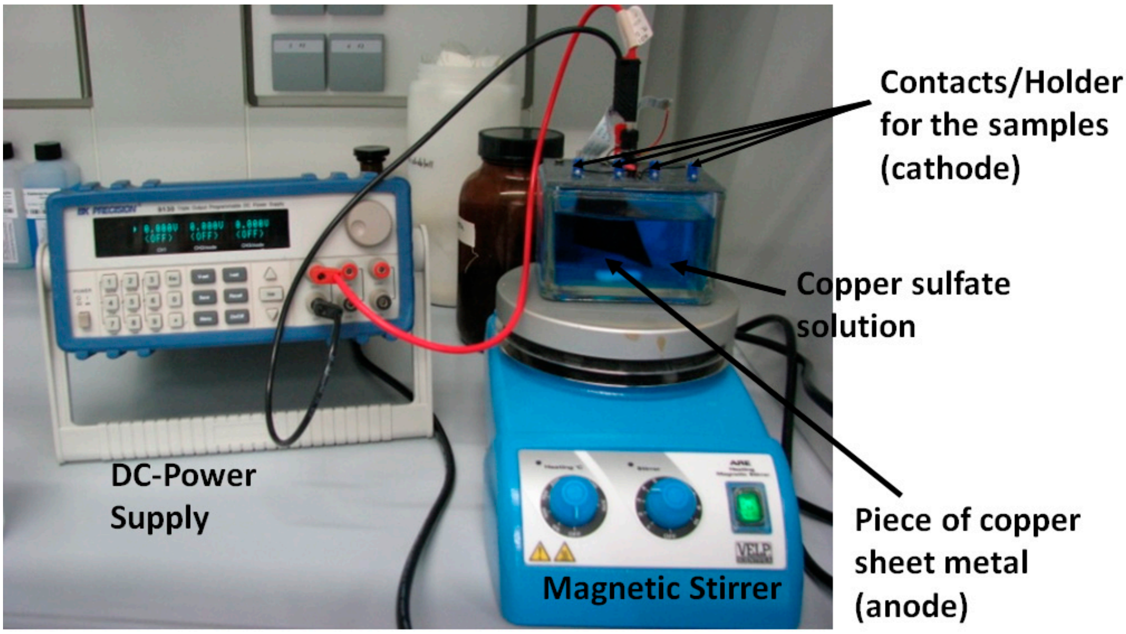

Figure S3. Custom electroplating setup. A glass vessel is filled with copper sulfate solution and covered with a top lid (air-tight). The lid contains plugs for the power supply and their corresponding connected holders for the anode (copper sheet metal) and the cathode (samples to be electroplated). A DC-power supply allows for adjusting voltage and amperage.

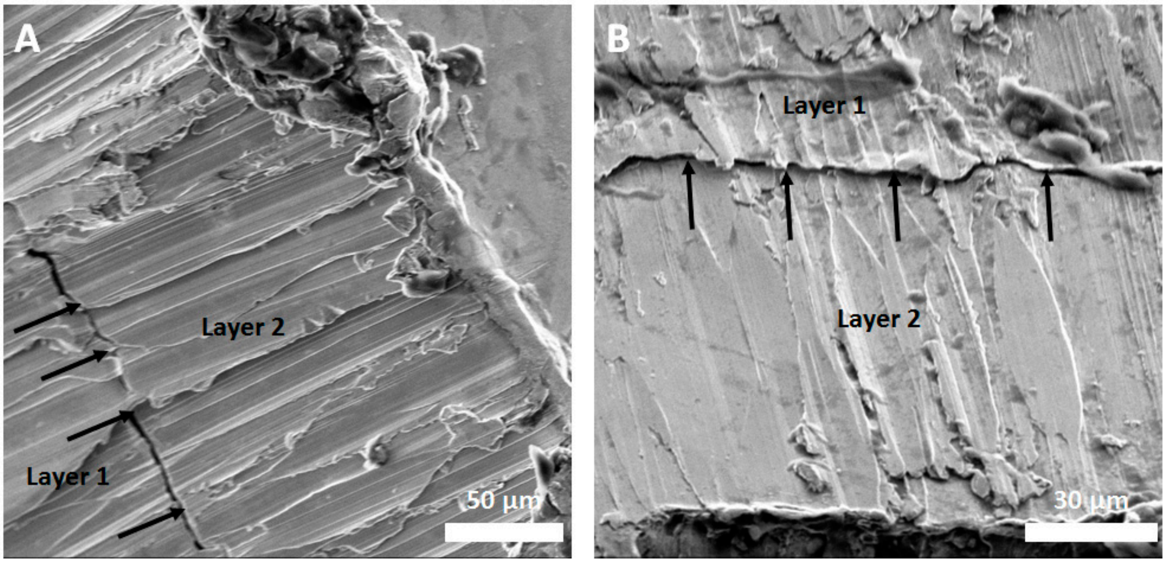

Figure S4. Representative SEM images of copper deposited on copper by electroplating**.** (**A**) and (**B**) show the cross-section of two different samples after cutting with the saw. A seam between the original material (Layer 1) and the deposited layer (Layer 2) can be clearly identified (indicated by black arrows).
